# Adrenal Angiomyolipoma: A Case Report

**DOI:** 10.7759/cureus.6881

**Published:** 2020-02-04

**Authors:** Mohamed Ahmed, Ahmed Mahmoud, Amarseen Mikael, Rasha Saeed, Dina Elias

**Affiliations:** 1 Surgery, University of California, Riverside, USA; 2 Surgery, Riverside Community Hospital, Riverside, USA; 3 Surgery, Arrowhead Regional Medical Center, Fontana, USA; 4 Trauma, Riverside Community Hospital, Riverside, USA

**Keywords:** angiomyolipoma, adrenal mass

## Abstract

Angiomyolipoma (AML) is a mesenchymal tumor that arises from perivascular epithelioid cells most commonly seen in the kidney and rarely in extrarenal sites. Adrenal AML is a rare clinical entity with only 16 cases reported in the literature according to the World Health Organization report in 2017. We present a 68-year-old male evaluated in our emergency room with worsening chronic right upper quadrant abdominal pain. Computerize tomography (CT) revealed a large right adrenal mass. Adrenelectomy was performed, and the histopathology report confirmed the diagnosis and the tumor size was the largest ever reported. Patient was discharged uneventfully.

## Introduction

Angiomyolipoma (AML) is a mesenchymal tumor derived from perivascular epithelioid cells and consist of blood vessels, muscle cells, and fat tissue [1]. Extrarenal AML is most commonly found to occur in the liver and less commonly in the spleen, lungs, retroperitoneum, bone, and ovaries [2-3]. Adrenal AML is very rare with only 16 reported worldwide, five of which in the English literature [4-5]. It is usually asymptomatic and incidentally diagnosed on a radiological investigation of the abdomen [6]. We present a case of right-sided giant adrenal AML found in a patient presented with right-sided abdominal pain.

## Case presentation

**Figure 1:**
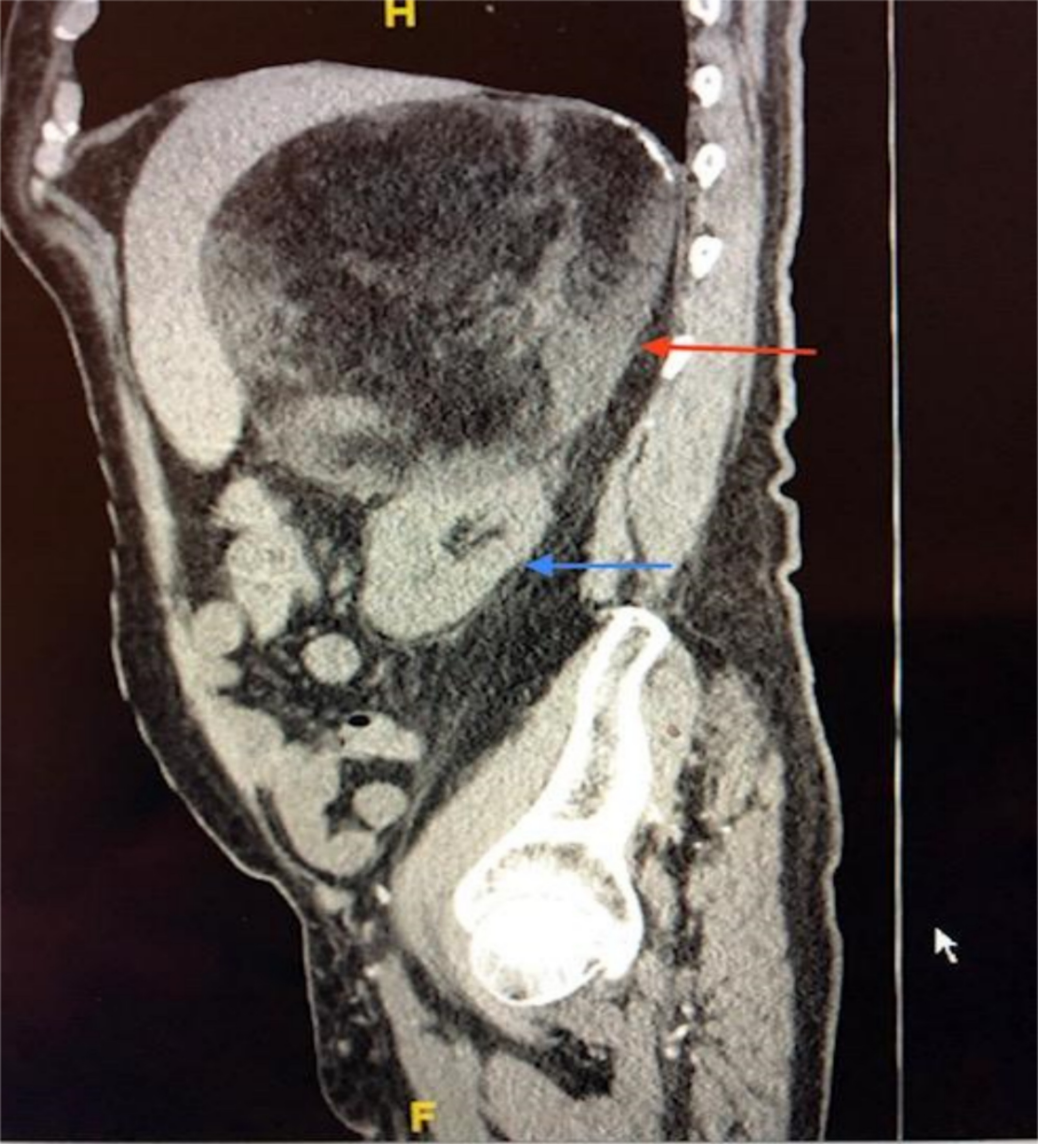
Computed tomography of the abdomen and pelvis Adrenal mass (red arrow); Kidney (blue arrow)

A 68-year-old male presented to our emergency room with acute on chronic right upper quadrant pain physical examination revealed a right upper quadrant mass with discomfort on deep palpation. Past medical history includes diabetes mellites, renal insufficiency, and hypertension requiring multiple medications to control. CT revealed a 17.3 x 14.6 x 16 cm fat-containing mass originating from the right adrenal gland (Figure 1).

**Figure 2:**
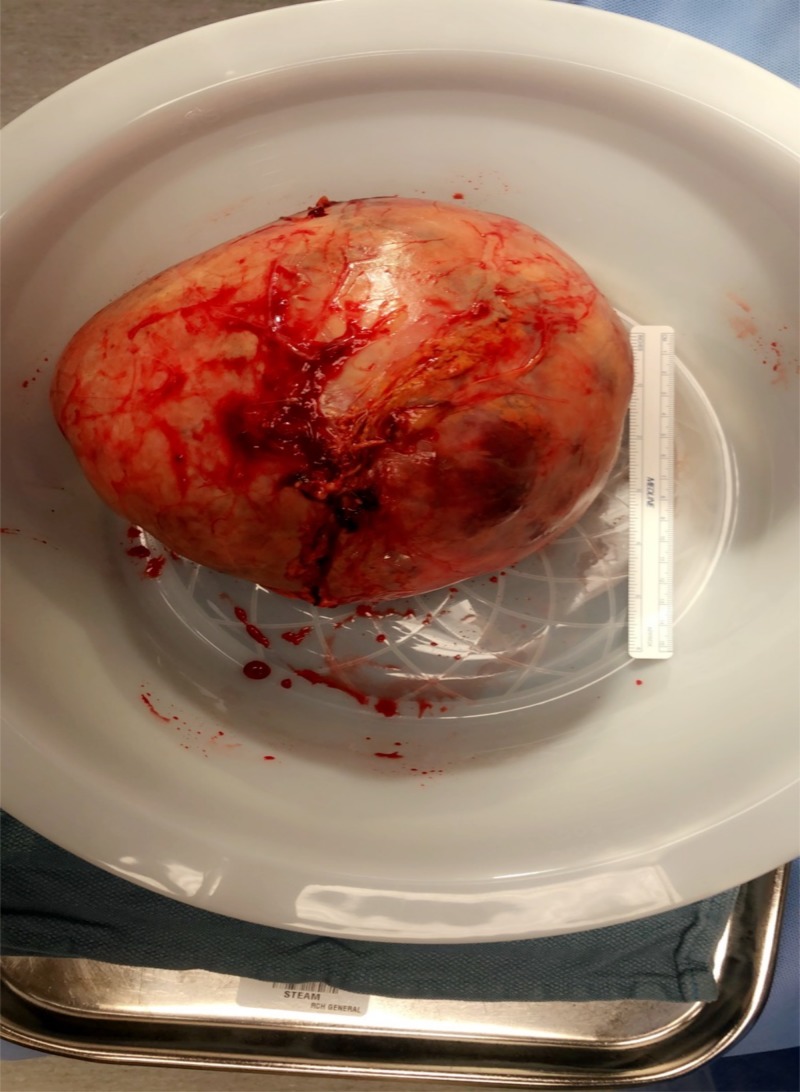
Adrenal gland tumor Large adrenal mass

Patient was admitted to the hospital and it was determined to be a nonfunctioning adrenal mass after a series of testing to include renin activity of 0.449 ng/ml/hr (0.167-5.380), aldosterone (1.0) ng/dL (0.0-30.0), cortisol 13.25 mcg/dL (3.1-22.4), ACTH 40.3 pg/mL (7.2-63.3), plasma metanephrine 12 pg/mL ( 0-62), plasma normetanephrine 104 pg/mL (0-145). Urine 24-h metanephrine 48 ug/24hr (45-290), urine 24-h normetanephrine 244 ug/24hr (82-500). Patient was taken to the operating room and a right adrenalectomy was performed (Figure 2).

**Figure 3:**
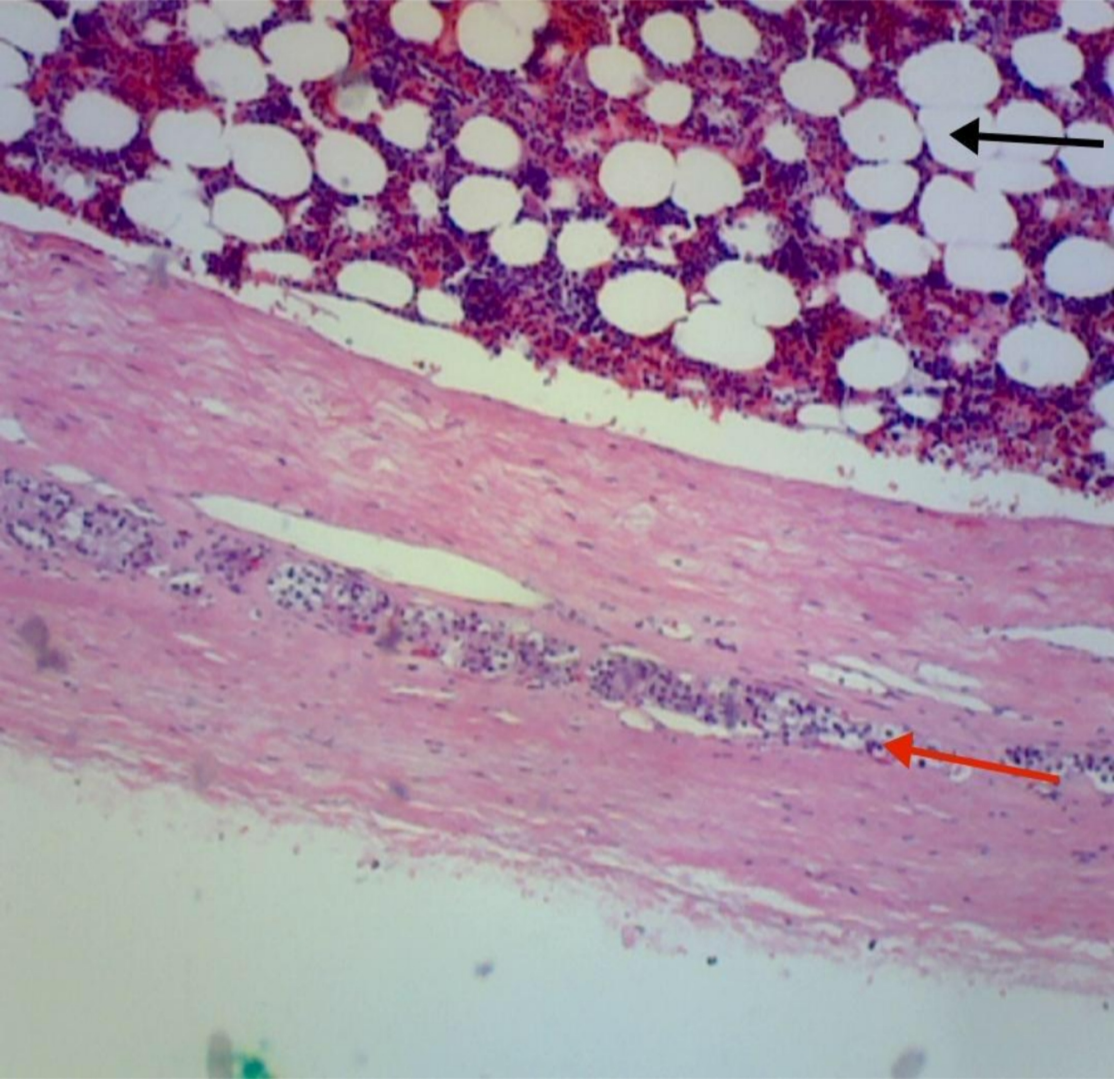
Histopathology Red arrow: Fibrous capsule with a thin band of compressed adrenal cortex. Black Arrow: Hematopoietic tissue.

Histopathology confirmed the diagnosis (Figure 3).

The patient had an uneventful recovery and was discharged from the hospital.

## Discussion

In 1951, Morgan described a benign renal tumor composed of blood vessels, muscle, and fat and the term "angiomyolipoma" was introduced [7]. The use of better imaging techniques has been associated with a surge of incidental adrenal lesions. Lipomatous tumors of the adrenal gland are uncommon and account for roughly 5% of primary adrenal tumors [8]. It includes myelolipoma, lipomas, teratoma, liposarcoma, and rarely AML [9]. Adrenal AML is an extremely uncommon tumor usually detected incidentally. Small asymptomatic tumors may be managed with active serial imaging surveillance, however, large asymptomatic AML should be removed to avoid spontaneous rupture due to the presence of abnormal elastin in the tumor [10]. Symptomatic tumor treatment includes excision, angioembolization, or cryotherapy. In our case, the tumor size was 20 x 16 cm and the largest ever reported was 15x16 cm [11].

## Conclusions

AML is a mesenchymal tumor usually found in the kidney. Extrarenal AML is uncommon, with the adrenal gland being an exceedingly rare location. They are often misdiagnosed as sarcomatoid carcinoma, carcinoma or sarcoma and are known to be diagnostic challenges to pathologists. The tumor can grow to a significant size, and we believe our case is the largest ever reported. An asymptomatic small tumor can be managed with active surveillance; however, large or symptomatic tumors should be resected to avoid complications including spontaneous rupture likely due to the presence of abnormal elastin. Angioembolization or cryotherapy are considered as other options of therapy.
